# The association between warfarin usage and international normalized ratio increase: systematic analysis of FDA Adverse Event Reporting System (FAERS)

**DOI:** 10.20517/jca.2023.33

**Published:** 2023-10-17

**Authors:** Robert Morris, Megan Todd, Nicole Zapata Aponte, Milagros Salcedo, Matthew Bruckner, Alfredo Suarez Garcia, Rachel Webb, Kun Bu, Weiru Han, Feng Cheng

**Affiliations:** 1 Department of Pharmaceutical Sciences, Taneja College of Pharmacy, University of South Florida, Tampa, FL 33612, USA.; 2 Department of Mathematics & Statistics, College of Art and Science, University of South Florida, Tampa, FL 33620, USA.; 3 Department of Biostatistics and Epidemiology, College of Public Health, University of South Florida, Tampa, FL 33612, USA.

**Keywords:** Warfarin, FAERS, FDA, pharmacovigilance, international normalized ratio, INR

## Abstract

**Introduction:**

Elevated international normalized ratio (INR) has been commonly reported as an adverse drug event (ADE) for patients taking warfarin for anticoagulant therapy.

**Aim:**

The purpose of this study was to determine the association between increased INR and the usage of warfarin by using the pharmacovigilance data from the FDA Adverse Event Reporting System (FAERS).

**Methods:**

The ADEs in patients who took warfarin (*N* = 77,010) were analyzed using FAERS data. Association rule mining was applied to identify warfarin-related ADEs that were most associated with elevated INR (*n* = 15,091) as well as possible drug-drug interactions (DDIs) associated with increased INR. Lift values were used to identify ADEs that were most commonly reported alongside elevated INR based on the correlation between both item sets. In addition, this study sought to determine if the increased INR risk was influenced by sex, age, temporal distribution, and geographic distribution and were reported as reporting odds ratios (RORs).

**Results:**

The top 5 ADEs most associated with increased INR in patients taking warfarin were decreased hemoglobin (lift = 2.31), drug interactions (lift = 1.88), hematuria (lift = 1.58), asthenia (lift = 1.44), and fall (lift = 1.32). INR risk increased as age increased, with individuals older than 80 having a 63% greater likelihood of elevated INR compared to those younger than 50. Males were 9% more likely to report increased INR as an ADE compared to females. Individuals taking warfarin concomitantly with at least one other drug were 43% more likely to report increased INR. The top 5 most frequently identified DDIs in patients taking warfarin and presenting with elevated INR were acetaminophen (lift = 1.81), ramipril (lift = 1.71), furosemide (lift = 1.64), bisoprolol (lift = 1.58), and simvastatin (lift = 1.58).

**Conclusion:**

The risk of elevated INR increased as patient age increased, particularly among those older than 80. Elevated INR frequently co-presented with decreased hemoglobin, drug interactions, hematuria, asthenia, and fall in patients taking warfarin. This effect may be less pronounced in women due to the procoagulatory effects of estrogen signaling. Multiple possible DDIs were identified, including acetaminophen, ramipril, and furosemide.

## Graphical Abstract

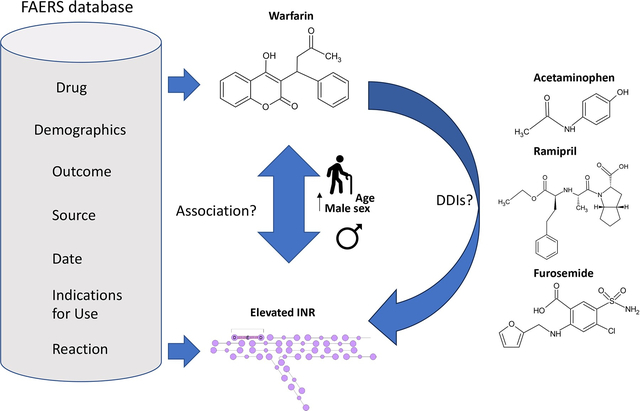

## INTRODUCTION

Warfarin, commercially sold under the name of Coumadin in the United States, is an anticoagulant drug frequently prescribed to treat blood clots, deep vein thrombosis, and strokes in patients presenting with atrial fibrillation or artificial heart valves^[1]^. Warfarin was first approved by the Food and Drug Administration (FDA) in the United States in 1954 for the treatment of blood clots and, as of 2019, is one of the most prescribed medications in the United States, with approximately 14 million prescriptions^[2]^. Formation of blood clots, termed thrombosis, may occur either in the arteries or veins and result in either partial or complete blockage depending on the size of the occlusion that is generated^[3]^. Many underlying cardiovascular risk factors including smoking status, pregnancy, atrial fibrillation, morbid obesity, and inflammation predispose individuals to greater risks of blood clot formation, with as many as 100,000 mortalities attributed to blood clotting events reported each year in the United States^[3]^. With the rise in prevalence of risk factors associated with an increased likelihood of blood clotting diagnosis and the high frequency in which warfarin is prescribed to treat these conditions, there is a growing necessity for further investigation of the mechanisms of warfarin’s advers effects and possible drug-drug interactions.

Warfarin functions as a potent vitamin K antagonist molecule that consequently stifles the generation of various clotting factors such as clotting factors II, VII, IX, and X, as well as several regulatory proteins involved in the clotting cascade, including protein C, protein Z, and protein S^[4]^. Many of the precursor clotting factors synthesized by the liver require the addition of vitamin K as a cofactor in order to bind to phospholipids located on the surface vascular endothelium and conduct the initiation of the clotting cascade^[5]^. Firstly, the glutamic acid residues of the precursor clotting factors undergo a gamma-carboxylation event mediated by the enzymatic activity of gamma-glutamyl carboxylase^[2,4]^. Carboxylation of the glutamic acid residues is coupled to the simultaneous conversion of reduced vitamin K hydroquinone to vitamin K epoxide, a reaction that must occur in order for the residues to undergo the carboxylation event^[2,5]^. The vitamin K epoxide can then subsequently be converted back to vitamin K and its reduced form through the reverse reaction, a process that is carried out by the enzyme vitamin K epoxide reductase (VKOR)^[5]^. Warfarin functions as a competitive inhibitor of the VKORC1 subunit of the enzyme that catalyzes this reverse reaction, preventing the replenishing of vitamin K supplies needed to activate hepatic clotting factors and facilitating their binding to blood vessels^[2]^. Consequently, warfarin aids in the prevention of the formation of adverse blood clots through stifling of proper clotting factor activity.

Monitoring of the efficacy of warfarin treatment and determination of appropriate dosing is critical to modulate the risk of severe bleeding with the benefit of decreased blood clotting risk. Prothrombin, also termed clotting factor II, is first converted into its active form, thrombin (factor IIA), and then catalyzes the activation of fibrinogen to fibrin in order to develop thromboses at sites of tissue damage^[6]^. Prothrombin time (PT) is one of the clinical standards for assessing the amount of time it takes for the patient’s blood to clot, measured in seconds, with high PT values associated with increased liver damage and decreased prothrombin secretion^[6,7]^. In addition, patients administered vitamin K antagonists (VKAs) such as warfarin for anticoagulation treatment are commonly assessed using PT as a means to quantify coagulation status^[8]^.

In order to allow for comparison between PT test results and account for variability in test reagents used that function differently as well as differences in instrument sensitivity, the PT is converted into the international normalized ratio (INR) by incorporating two correction factors, the mean normal PT and an international sensitivity index (ISI) value that ranges in value from 0.9 to 1.7^[8,9]^. For patients not administered anticoagulation therapy, a physiologically normal INR value is approximately 1; however, the therapeutic dosage of VKAs for patients on blood thinners ranges between 2 and 3^[7,9]^. Many factors may influence a patient’s INR reading and response to anticoagulation treatment, including possible drug interactions with salicylates such as aspirin and the antiarrhythmic drug amiodarone, smoking status, diet, herbal supplements, and vitamin K consumption^[9,10]^. Consequently, dose efficacy is evaluated every 3–4 weeks for patients on anticoagulation therapy^[9]^.

Although the effects of warfarin on INR are well known, additional factors and confounding variables that influence warfarin’s impacts on patient INR are not well characterized. Patients taking Warfarin for anticoagulant therapy report many adverse drug events including skin necrosis, vomiting, hemorrhaging, anemia, stomach pain, joint pain, and numbness^[9,11]^. In this study, data from the FDA Adverse Event Reporting System (FAERS) was used to identify frequent side effects reported for patients taking warfarin that are most associated with an increase in patient INR. FAERS is the largest open-access drug event repository in the world, with approximately 26 million reports currently stored in the database, and at least 1 million new reports are added each year by patients, drug manufacturers, medical professionals, and manuscript publications^[12–14]^. Their online reporting system, MedWatch, was created to make it easier for consumers and health professionals to report adverse events themselves^[15]^. This program helps support the FDA post-marketing safety surveillance program, as adverse events to medications and biologics may happen even after FDA approval. After data collection through MedWatch, the information is presented in the FDA Adverse Events Reporting System, FAERS^[15]^.

For this study, association rule mining (ARM) was utilized in order to find strong correlations between warfarin-related adverse drug events (ADEs) and increased INR. In addition, ARM was to determine significant associations between elevated INR and different drugs taken concurrently with warfarin for anticoagulant therapy. Finally, additional confounders including age, gender, geographic distribution, and temporal distribution of reports were assessed.

## MATERIAL AND METHODS

The public FAERS dashboard was utilized to search and download FDA FAERS records. These records taken from the FAERS dashboard contain the following seven groups of relevant information: (1) drug information; (2) drug-related adverse events; (3) resulting patient outcome for the reported adverse event; (4) patient demographic information including body weight, sex, and patient age; (5) reporting source of the adverse event; (6) date of report submission; and (7) drug indications for use. Adverse drug events (ADEs) that were suspected to be related to Warfarin use by using five keyword search terms including “Warfarin”, “Jantoven”, “Coumadin”, “Warfarin sodium”, and “Warfarin potassium”. The “suspect product names” or “suspect product active ingredients” of a record that contains those generic or brand names of the drug (or salt of the drug) will be outputted from FAERS dashboard.

The majority of FAERS records in the public database are submitted to the FDA by either a health professional, directly by the patient, or from a pharmaceutical company. In addition, some records are extracted indirectly from publications. In this study, records extracted from publications were excluded for three primary reasons. First, these indirect reports are often duplicated in the FAERS database as they are reported by multiple companies or manufacturers, resulting in some reports having 2–10 duplicated copies in the database. The inclusion of these duplicated reports would inflate effect sizes and result in more false positives in downstream analysis. Next, these reports were filtered out as different conclusions may be drawn from different companies analyzing the literature, resulting in inconsistent ADE reports. Finally, these duplicate records only comprise approximately 2%–3% of the total number of reports available in the FAERS database. Thus, the removal of these duplicate records will have a negligible impact on the final conclusions.

A disproportionality analysis was conducted to compare the risk of elevated INR among the group of interest (experimental group) and the control group. The reporting odds ratio (ROR) for this comparison was subsequently calculated. A lower bound value greater than 1.0 for the 95% confidence interval was indicative of a statistically significantly greater likelihood of reporting elevated INR in the experimental group than in the control group. In addition, possible confounders include sex, age, country of origin, temporal distribution of reports, and patient outcome using RORs as to their effects on the risk of elevated INR. Data analysis was performed using R statistical software. For association rule mining, the following formulas were used in order to calculate the lift as well as the confidence and support values.

$$\text{Support}\;(\{X\}\to \{Y\})=\frac{\;\text{Number}\;\text{of}\;\text{reports}\;\text{containing}\;\text{both}\;X\;\text{and}\;Y}{\;\text{Total}\;\text{number}\;\text{of}\;\text{reports}\;}$$

$$\text{Confidence}\;(\{X\}\to \{Y\})=\frac{\;\text{Number}\;\text{of}\;\text{reports}\;\text{containing}\;\text{both}\;X\;\text{and}\;Y}{\;\text{Number}\;\text{of}\;\text{reports}\;\text{containing}\;X}$$

$$Lift(\{X\}\to \{Y\})=\frac{\;\text{Support}\;}{\;\text{Support}\;\left(X\right)\;*\;\text{Support}\;(Y)}$$

## RESULTS

### Top 15 ADEs related to warfarin use

The top 15 reported adverse drug events associated with warfarin usage that were reported to the FAERS database are presented in Table 1. Of the 77,010 total reports submitted for patients taking warfarin, the most frequently reported adverse event was increased INR (*n* = 15,091), accounting for approximately 20% of all reported adverse events. Other frequently reported ADEs comprising the top 5 for patients treated with warfarin included drug interactions (*n* = 8,041), hemorrhaging (*n* = 5,794), gastrointestinal hemorrhaging (*n* = 5,397), and decreased prothrombin levels (*n* = 5,040). Many of these commonly reported ADEs, such as anemia (*n* = 4,226), epistaxis (*n* = 3,023), hematuria (*n* = 2,693), contusions (*n* = 2,294), and decreased hemoglobin (*n* = 2,258), may be consequences of bleeding events frequently associated with warfarin use.

### ADEs associated with INR increase

Association rule mining was first used to identify ADEs that most frequently co-presented alongside increased INR in patients treated with warfarin. A lift value greater than 1 indicates that increased INR and the selected adverse drug event were present together in numbers greater than the expected number of instances. Among the assessed adverse drug events, the following exhibited a lift value greater than 1: decreased hemoglobin levels (lift = 2.31), drug interactions (lift = 1.88), hematuria (lift = 1.58), asthenia (lift = 1.44), fall (lift = 1.32), epistaxis (lift = 1.31), dyspnea (lift = 1.24), anemia (lift = 1.22), and gastrointestinal hemorrhage (lift = 1.07). Thus, elevated INR is frequently co-presented with multiple bleeding-related adverse events [Table 2].

### Temporal distribution of reported increased INR cases

**Figure 1.**
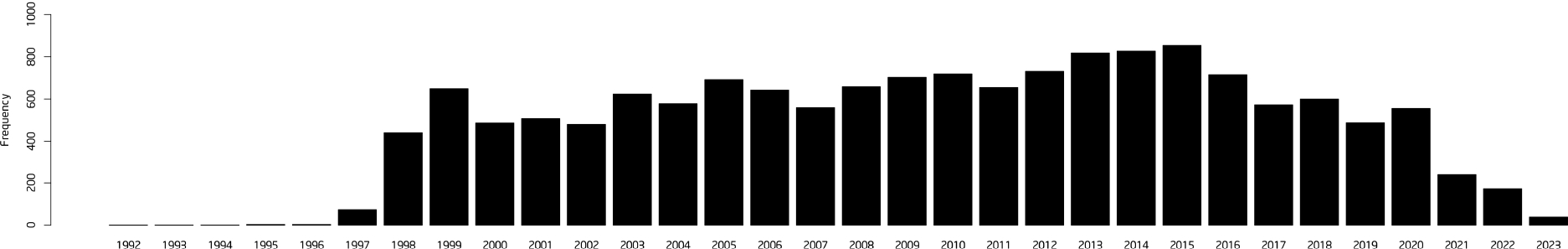
Temporal distribution of increased INR reports to the FAERS database.

Records from the FAERS database were collected between 1997 and 2023 in order to identify trends in the reported number of increased INR cases for patients treated with warfarin [Figure 1]. The number of reported instances of increased INR by individuals treated with warfarin for blood clots increased significantly between 1997 and 1999, demonstrating an approximately 6-fold increase in frequency from 100 reports in 1997 to over 600 reports in 1999. Between 1999 and 2015, the number of reports gradually increased to 800, prior to declining each year between 2015 and 2023.

### Geographical distribution of reported increased INR cases

**Figure 2.**
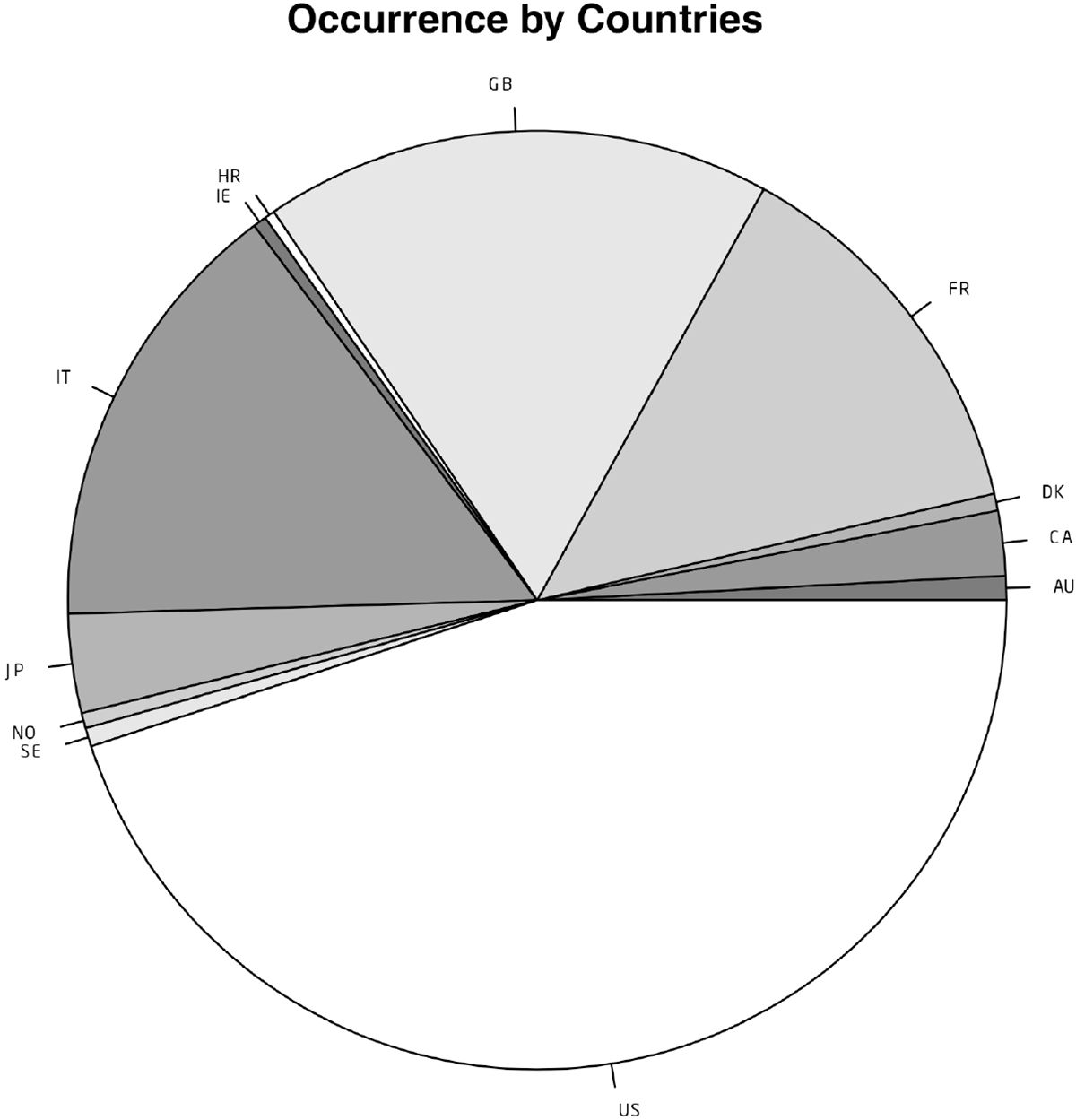
Geographical distribution of increased INR reports (case number > = 20) to the FAERS database.

Next, the geographical distribution of increased INR reports submitted for patients treated with warfarin was investigated and is presented in Figure 2. Reports in which the country of origin was not specified were excluded from this portion of the analysis. Approximately 45% of all elevated INR reports submitted to the FAERS database between 1997 and 2023 originated from the United States, the first country in which warfarin was granted government approval for the treatment of blood clots in humans in 1954. Reports originating from Great Britain, Italy and France accounted for another 45% of all reported cases, while the remaining reports were made for patients primarily in Japan, Canada, and Australia. These findings indicate that the possible association between warfarin and increased INR is a globally observed event, not one that is confounded by the geographical origins of the report.

### Age difference in the risk of increased INR

The role of age as a possible confounding variable of the association between warfarin treatment and increased INR was investigated. As shown in Table 3, patients in the 50 <= Age <= 70 demographic had a 24% greater likelihood (ROR = 1.24, 95%CI: 1.15–1.33) of reporting increased INR to the FAERS database than individuals younger than 50 years of age. Patients between the ages of 70 and 80 were 31% more likely than the control group (ROR = 1.31, 95%CI: 1.22–1.41), while individuals older than 80 years of age had 63% greater odds (ROR = 1.63, 95%CI: 1.52–1.76) than those younger than 50 years of age. Thus, there was a positive correlation between age and the risk of having an elevated INR.

### Sex difference in the risk of increased INR

The sex difference in the risk of increased INR treated with warfarin was assessed [Table 4]. Among the 32,477 female patients treated with warfarin, 6,317 reported an increased INR, while among the 37,886 male patients, 7,913 reported an increased INR. Males were 9% more likely to present with increased INR (ROR = 1.09, 95%CI: 1.05–1.14) than females in this sample population.

### Possible drug interactions related to increased INR

To detect whether the drug-drug interactions (DDIs) play an important role in the risk of increased INR, the frequency of increased INR in patients taking warfarin alone was compared with patients taking warfarin in combination with other drugs. As shown in Table 5, we observed 11,238 cases out of 52,756 individuals when considering patients who were administered warfarin while taking other drugs. Individuals taking warfarin concurrently with at least one other drug had 43% greater odds (ROR = 1.43, 95%CI: 1.38–1.49) of reporting increased INR relative to those taking warfarin exclusively (3,853 cases out of 24,254 individuals).

The association rule was then applied to identify which drug compounds may interact with warfarin. As shown in Table 6, the top 5 most frequently identified drug-drug interactions in patients taking warfarin and presenting with elevated INR were acetaminophen (lift = 1.81), ramipril (lift = 1.71), furosemide (lift = 1.64), bisoprolol (lift = 1.58), and simvastatin (lift = 1.58). Additional drugs that frequently co-occurred with high INR in patients taking warfarin include potassium chloride (lift = 1.58), omeprazole (lift = 1.55), levothyroxine (lift = 1.49), prednisone (lift = 1.45), metoprolol (lift = 1.45), lisinopril (lift = 1.44), atenolol (lift = 1.43), digoxin (lift = 1.43), atorvastatin (lift = 1.43), and aspirin (lift = 1.33).

## DISCUSSION

In this study, a systematic approach was used to assess the most prevalent adverse drug events reported by patients administered warfarin as well as important covariates that moderate the association between warfarin use and the risk of elevated INR. In addition, ARM was used to determine which adverse drug events of warfarin usage were most strongly associated with elevated INR and which drug-drug interactions were correlated most strongly with high INR readings. Decreased hemoglobin (lift = 2.31), drug interactions (lift = 1.88), hematuria (lift = 1.58), and asthenia (lift = 1.44) were the reported adverse drug events most associated with elevated INR. A post-hoc analysis (*n* = 6,536) of the J-RHYTHM Registry found a significant association between declining hemoglobin (< 12.0 g/dL) and the incidence of major hemorrhaging events (*P* = 0.004) in Japanese patients with non-valvular atrial fibrillation^[16]^. Hematuria is a common adverse drug event reported by those taking warfarin and has previously been shown to be significantly associated with supratherapeutic INR levels in a prospective observational study of 150 elderly receiving warfarin for anticoagulant therapy^[17]^. Finally, supratherapeutic INR is indicative of extremely slow blood clotting and elevated risk of internal bleeding, which may lead to inadequate supplies of oxygen for muscle function and contribute to the development of significant weakness and fatigue^[18]^.

Multiple covariates including age, gender, temporal distribution of reports, and report country of origin were analyzed with regard to their possible effects on the risk of elevated INR. Physiological changes in hemostasis and clotting factor expression occur with advancing age, promoting a procoagulatory state that increases an individual’s risk of having elevated INR^[19,20]^. As age increases, the synthesis of procoagulatory factors such as fibrinogen, factor FVIII, and factor FVII, as well as the formation of thrombin and platelet activation, also increases^[19]^. Conversely, the levels and efficacy of natural anticoagulants such as antithrombins have been shown to decline with age^[21]^. A case-control study assessing factors that influence INR values greater than 5 in elderly patients treated with warfarin (*n* = 304) found that the risk of elevated INR > 5 increased by 60% (RR = 1.60, 95%CI: 1.12–2.28) for every 10-year increase in age^[22]^. Women are less susceptible to elevated INR as the major sex hormone estrogen has been shown to promote the expression of procoagulatory factors including FVIII, FVII, FXII, von Willebrand Factor (VWF), and fibrinogen while simultaneously inhibiting the release of anticoagulatory factors such as protein S, antithrombin, and plasminogen activator inhibitor 1 (PAI-1)^[23,24]^. Because of this, females have higher coagulation capacity than males of comparable age and are less at risk of excessive bleeding and elevated INR while also being at greater risk for blood clots and thrombosis^[23,24]^.

The effects of country of patient origin may be largely influenced by polymorphisms in two genes, *CYP2C9* and *VKORC1*, that are unevenly distributed across racial and ethnic groups. The *VKORC1* gene encodes the primary target of warfarin, vitamin K epoxide reductase, which regulates the rate-limiting step of converting vitamin K epoxide to vitamin K^[25]^. A commonly occurring non-coding mutation in the promoter region of *VKORC1,* which is estimated to be present in 90% of Asian populations, 40% of Caucasians and 14% of African Americans, termed VKORC1, c.−1639G>A is associated with greater patient sensitivity to warfarin and necessitates administration of lower doses^[25,26]^. CYP2C9 is the primary metabolizer of warfarin, particularly the more potent S-enantiomer, and the *CYP2C9* alleles CYP2C9*2 and CYP2C9*3 are well-characterized variants that promote increased warfarin sensitivity due to reduced enzymatic function^[25,26]^. These alleles are present in 10%–20% of Caucasians and explain a large amount of the variation in warfarin response among this population; however, these alleles are considerably less common in populations of African descent (0%–6%) and other variants are responsible for conferring difference degrees of warfarin sensitivity^[25,26]^. Thus, genetic composition and country of origin may influence the risk of elevated INR in patients taking warfarin.

Between 2015 and 2022, the total number of warfarin reports submitted to the FAERS database began to decline. This decline in submitted reports may be due to several reasons. First, the emergence and FDA approval of the first direct oral anticoagulant (DOAC) dabigatran in 2010 and the subsequent approval of two additional drugs in this class, edoxaban and betrixaban, in 2015 and 2017, respectively, provided new options for the treatment of thromboembolic events^[27,28]^. By the end of 2017, all Medicare Prescription Coverage Plans covered the costs for at least 1 DOAC, making them readily available for most patients^[28]^. In addition, they became more popular as a frontline treatment for thromboembolisms beginning in 2013 and were more frequently prescribed by medical providers due to their perceived safety compared to warfarin^[28,29]^. A retrospective study conducted on data collected from the Medicare Provider Utilization and Payment database (*n* = 325,666) found that the DOAC prescription volume increased from 14.1% of all anticoagulant prescriptions in 2013 to 57.3% of all anticoagulant prescriptions in 2018^[29]^. A systematic review of the literature using phase 3 clinical trial data comparing DOAC to warfarin usage found a 20% lower likelihood of any stroke (OR = 0.80, 95%CI: 0.73–0.88), 44% lower risk of systemic embolism (OR = 0.56, 95%CI: 0.34–0.93), and a 11% decrease in likelihood of total mortality (OR = 0.89, 95%CI: 0.84–0.95)^[30]^. Finally, 2016 guidelines published by the American College of Chest Physicians (CHEST) and 2019 guidelines modifications published by the American College of Cardiology/American Heart Association/Heart Rhythm Society (ACC/AHA/HRS) formally recommend DOACs as the primary anticoagulation therapy over warfarin^[28]^. Therefore, warfarin usage may have declined due to the availability of safer alternatives for anticoagulation therapy.

The issue of DDIs is significant when using warfarin^[31–33]^. Drug interactions may increase the risk of bleeding for warfarin patients. Using association rules, many possible drug-drug interactions were identified in patients taking warfarin with regard to INR risk in this paper. Acetaminophen is an over-the-counter painkiller that has previously been shown to be independently associated with elevated INR and decreased production of vitamin K-dependent factors FVII and FIX^[34,35]^. Further studies have demonstrated that the metabolite of acetaminophen N-acetyl-p-benzoquinone-imine (NAPQ1) inhibits multiple components of the vitamin K cycle, including promoting the oxidation of vitamin K-hydroquinone (KH2), stifling vitamin K-dependent carboxylation, and inhibiting the functionality of vitamin K-epoxide reductase (VKOR)^[34,36]^. Thus, acetaminophen may act synergistically with warfarin to potentiate the anticoagulatory properties of warfarin. ACE inhibitors such as ramipril and lisinopril are used to treat hypertension by inhibiting the production of angiotensin II, a protein involved in the constriction of blood vessels^[37]^. Previous studies have shown that patients treated with ACE inhibitors such as lisinopril and fosinopril had decreased levels of the procoagulatory factors including fibrinogen, PAI-1, and VWF^[38–40]^. In addition, patients who were randomly assigned to a lisinopril-based therapy regimen had a 27% higher risk (HR = 1.27, 95%CI: 1.06–1.51) of being hospitalized for severe GI bleeding compared to those taking the calcium channel blocker amlodipine^[40]^. Thus, ACE inhibitors may work in concert with warfarin to suppress the production and activity of procoagulatory factors, thus contributing to increased INR and greater bleeding risk.

Loop diuretics such as furosemide are commonly administered to individuals presenting with fluid overload symptoms such as heart failure or kidney disease and function by reducing the reabsorption of salt by kidney tissue^[41]^. In many cases, this may result in dehydration, which has been shown to promote clot lysis (hypercoagulable state) and elevate PT^[36]^. Thus, warfarin and furosemide may work synergistically in elevating INR by inhibiting the synthesis of clotting factors and accelerating the premature dissolution of clots, respectively. Beta-blockers such as atenolol and metoprolol block the binding of epinephrine and norepinephrine to B1 beta-adrenoreceptors, resulting in slowed heart rate and vasodilation of arterial vessels^[42]^. A retrospective analysis of patients presenting with chronic heart failure from the Department of Veteran Affairs (VA) who are taking warfarin (*n* = 66,988) found that those taking atenolol had a 27% greater risk (HR = 1.27, 95%CI: 1.18–1.38) of a future hemorrhagic event and those administered metoprolol had 38% greater risk (HR = 1.38, 95%CI: 1.28–1.48) compared to those taking carvedilol^[43]^. However, in the same study, no significant effects on INR were observed in a subpopulation of the original dataset (*n* = 3,546)^[43]^. The literature concerning the association between increased PT and the use of beta blockers has been mixed. Some studies suggest no association between the use of beta blockers and increased PT^[43–45]^. Conversely, adrenergic receptors have been shown to stimulate the sympathetic nervous system and promote the synthesis of blood clotting factors as well as factors involved in the fibrinolysis pathway^[46]^. Thus, beta-blockers may work antagonistically to warfarin to modulate blood thinning, but this may be a secondary passive effect that is not enough to counteract the INR-increasing effects of warfarin in some patients. Finally, statins such as simvastatin and atorvastatin have been shown to interact with warfarin by delaying warfarin metabolism and potentiating its effects and have been shown to be associated with increased INR^[47,48]^.

Despite these promising findings, there are some limitations. First, due to the observational nature of this study, we cannot effectively draw causative conclusions between the observed associated ADEs and increased INR. In addition, we can only state that these proposed drug-drug interactions are associated with increases in INR. Next, some drug-drug interactions discussed are not well characterized in the literature and are postulated based on drug mechanisms of action. Conflicting reports also obfuscate the true severity of these possible drug-drug interactions. Thus, we cannot state that these drug-drug interactions have clinical relevancy without clinical assessment. Future studies will focus on validating these possible drug-drug interactions and identifying additional drug-drug interactions using the FAERS database as new reports are added. Finally, as is the case with observational studies of this nature, there is potential bias due to the under-reporting of ADEs to FAERS. However, the effects of this type of bias should be relatively minimal as INR measurements are frequently made for patients taking warfarin (usually monthly or bimonthly) and are a standardized part of all warfarin patient checkups. Moreover, since INR can be conveniently measured at home, there should be minimal adverse effects of this association due to under-reporting.

In conclusion, patients prescribed warfarin were at greater risk of reporting an increase in elevated INR. This effect may be less pronounced in women due to the procoagulatory effects of estrogen signaling. The top 5 ADEs most associated with increased INR in patients taking warfarin were decreased hemoglobin, drug interactions, hematuria, asthenia, and fall. INR risk increased as age increased, with individuals older than 80 having a 63% greater likelihood of elevated INR compared to those younger than 50. Males were 9% more likely to report increased INR as an ADE compared to females. Patients prescribed warfarin and at least one other drug were at greater risk of reporting an increase in INR than those taking warfarin only. The top 5 most frequently identified drug-drug interactions in patients taking warfarin and presenting with elevated INR were acetaminophen (lift = 1.81), ramipril (lift = 1.71), furosemide (lift = 1.64), bisoprolol (lift = 1.58), and simvastatin (lift = 1.58). Many drugs may amplify the anticoagulation effects of warfarin by delaying warfarin metabolism, increasing the production of anticoagulant factors, or decreasing the synthesis of procoagulatory factors.

## Figures and Tables

**Table 1.** Top 15 ADEs of warfarin from the FAERS database as reported through 2023

	Adverse events	Case number

1	International normalized ratio increased	15,091
2	Drug interaction	8,041
3	Hemorrhage	5,794
4	Gastrointestinal hemorrhage	5,397
5	Prothrombin level decreased	5,040
6	Anemia	4,226
7	Epistaxis	3,023
8	Fall	2,937
9	Hematuria	2,693
10	Drug ineffective	2,520
11	International normalized ratio decreased	2,466
12	Dyspnea	2,452
13	Contusion	2,294
14	Hemoglobin decreased	2,258
15	Asthenia	2,214

**Table 2.** Association between INR increase and other ADRs in patients prescribed Warfarin

Association rule	Count	Support	Confidence	Coverage	Lift

{hemoglobin decreased=1} -> {INR increase=1}	1021	0.0133	0.452	0.0294	2.31
{drug interaction=1} -> {INR increase=1}	3166	0.0411	0.368	0.112	1.88
{hematuria=1} -> {INR increase=1}	834	0.0108	0.309	0.0350	1.58
{asthenia=1} -> {INR increase=1}	634	0.00823	0.282	0.0291	1.44
{fall=1} -> {INR increase=1}	759	0.00986	0.259	0.0381	1.32
{epistaxis =1} -> {INR increase=1}	775	0.0101	0.256	0.0393	1.31
{dyspnea=1} -> {INR increase=1}	650	0.00844	0.243	0.0347	1.24
{anemia=1} -> {INR increase=1}	1231	0.0160	0.241	0.0664	1.22
{gastrointestinal hemorrhage=1} -> {INR increase=1}	1396	0.0181	0.210	0.0861	1.07
{contusion=1} -> {INR increase=1}	470	0.00610	0.203	0.0301	1.04
{hemorrhage=1} -> {INR increase=1}	4509	0.0585	0.200	0.293	1.02

**Table 3.** Age difference in the risk of increased international normalized ratio

	With INR increased	Without INR increased	ROR (95%CI) & *P*-value

Age < 50	1,142	5,107	
50 <= Age <= 70	4,458	16,072	1.24 (1.15~1.33), *P* < 0.0001
70 < Age <= 80	4,030	13,786	1.31 (1.22~1.41), *P* < 0.0001
Age > 80	3,667	10,037	1.63 (1.52~1.76), *P* < 0.0001

**Table 4.** Sex difference in the risk of increased international normalized ratio

	With INR increased	Without INR increased	ROR (95%CI) & *P*-value

Female	6,317	26,160	
Male	7,913	29,973	1.09 (1.05 to 1.14), *P*-value < 0.0001

**Table 5.** Compare table between all and single drug use

	With INR increased	Without INR increased	ROR (95%CI) & *P*-value

Single cases	3,853	20,401	
Drug combination	11,238	41,518	1.43 (1.38 to 1.49), *P*-value < 0.0001

**Table 6.** Association rules to identify which compounds may interact with warfarin

Association rule	Count	Support	Confidence	Coverage	Lift

{acetaminophen=1} -> {INR increased=1}	800	0.0104	0.355	0.0293	1.81
{ramipril=1} -> {INR increased=1}	441	0.00573	0.335	0.0171	1.71
{furosemide=1} -> {INR increased=1}	1549	0.0201	0.321	0.0626	1.64
{bisoprolol =1} -> {INR increased=1}	846	0.0110	0.310	0.0355	1.58
{simvastatin=1} -> {INR increased=1}	666	0.00864	0.311	0.0279	1.58
{potassium chloride =1} -> {INR increased=1}	508	0.00660	0.309	0.0214	1.58
{omeprazole=1} -> {INR increased=1}	793	0.0103	0.303	0.0340	1.55
{levothyroxine=1} -> {INR increased=1}	621	0.0081	0.292	0.0276	1.49
{prednisone=1} -> {INR increased=1}	407	0.00529	0.284	0.0186	1.45
{metoprolol=1} -> {INR increased=1}	942	0.01223	0.283	0.0431	1.45
{lisinopril=1} -> {INR increased=1}	662	0.00860	0.282	0.0304	1.44
{atenolol=1} -> {INR increased=1}	450	0.0584	0.280	0.0209	1.43
{digoxin=1} -> {INR increased=1}	1118	0.0145	0.280	0.0519	1.43
{atorvastatin=1} -> {INR increased=1}	536	0.0070	0.280	0.0248	1.43
{aspirin=1} -> {INR increased=1}	1024	0.0132	0.260	0.0512	1.33
